# IMPLEMENTATION OF AN EDUCATION INTERVENTION FOR MULTIPLE MYELOMA PATIENTS RECEIVING STEM CELL TRANSPLANT

**DOI:** 10.1093/geroni/igac059.933

**Published:** 2022-12-20

**Authors:** Sean Halpin, Michael Konomos

**Affiliations:** Evidera, Decatur, Georgia, United States; Emory University, Atlanta, Georgia, United States

## Abstract

Older adults have an increased risk for multiple myeloma (MM), yet they are often unable to collect sufficient healthy stem cells for autologous stem cell transplant (ASCT). We sought to assess the implementation of educational intervention videos, termed Ready for Transplant (R4T), for MM patients both during the 18-month study and at 12 and 18-months post-study. Guided by the Reach, Effectiveness, Adoption, Implementation and Maintenance (RE-AIM) framework, we observed 152 clinical encounters for 70 patients who received the intervention. Patients who reported viewing the R4T videos (56%) tended to be older and male and were more likely to admit for transplant than those who did not report viewing the videos (risk ratio, 2.3; 95% CI, 0.61, 3.88). At 12- and 18-months-post, nurses reported still using the R4T videos and found them particularly useful during times when patients were unable to attend the hospital for in-person visits due to the pandemic.

